# Gender differences in the prevalence of nonalcoholic fatty liver disease in the Northeast of Thailand: A population-based cross-sectional study

**DOI:** 10.12688/f1000research.12417.2

**Published:** 2017-10-20

**Authors:** Ueamporn Summart, Bandit Thinkhamrop, Nittaya Chamadol, Narong Khuntikeo, Metha Songthamwat, Christina Sunyoung Kim

**Affiliations:** 1 Faculty of Public Health, Khon Kaen University, Khon Kaen, 40002, Thailand; 2 Department of Epidemiology and Biostatistics, Faculty of Public Health, Khon Kaen University, Khon Kaen, 40002, Thailand; 3 Cholangiocarcinoma Screening and Care Program (CASCAP), Khon Kaen University, Khon Kaen, 40002, Thailand; 4 Department of Radiology, Faculty of Medicine, Khon Kaen University, Khon Kaen, 40002, Thailand; 5 Department of Surgery, Faculty of Medicine, Khon Kaen University, Khon Kaen, 40002, Thailand; 6 Department of Obstetrics and Gynecology, Udonthani Regional Hospital, Udonthani, 41000, Thailand

**Keywords:** Nonalcoholic fatty liver disease, Gender differences, Post-menopausal, Ultrasonography, Asian population

## Abstract

**Background.** Nonalcoholic fatty liver disease (NAFLD) is the leading cause of chronic liver disease. A large number of studies have strongly described larger proportions of men being afflicted with NAFLD than women; however, recent studies investigating the role of gender and NAFLD have exposed the contrary.

**Methods. **This cross-sectional study utilized data from the baseline survey of an ongoing cohort study called the Cholangiocarcinoma Screening and Care Program (CASCAP), conducted in the northeastern region of Thailand between March 2013 and September 2015. Information regarding socio-demographic, including gender, was collected using a standardized self-administered questionnaire. NAFLD was diagnosed with ultrasonography by board-certified radiologists. A binomial regression was used for estimating the prevalence differences, odds ratios (OR) and the 95% confidence intervals (CI) of NAFLD between men and women.

**Results.** A total of 34,709 participants (27,073 females and 7,636 males) were recruited. The prevalence of NAFLD in women was 22.9% (95% CI: 22.5 to 23.5), whereas it was only 18.3% (95% CI: 17.4 to 19.2) in men. After adjusting for age and presence of diabetes mellitus and other underlying diseases, the prevalence was significantly higher in women, with adjusted prevalence difference of 4.2% (95% CI: 3.2 to 5.2) and adjusted OR of 1.3 (95% CI: 1.2 to 1.4). Women had a higher prevalence of NAFLD than men in all age groups and the largest difference was found in those aged 56-60 years (prevalence = 27.4% versus 21.2%; adjusted prevalence difference = 9.4%; 95% CI: 7.9 to 10.9; adjusted OR = 1.8; 95% CI: 1.8 to 2.0).

**Conclusion.** NAFLD is more likely to affect women more than men, in particular, among the population 56-60 years of age, which is the post-menopausal transitional period. Therefore, post-menopausal women should be the target for interventions or further investigation for NAFLD.

## Introduction

Nonalcoholic fatty liver disease (NAFLD) is the leading cause of chronic liver disease and a major public health problem worldwide ^1^. Its prevalence is increasing globally, and is currently estimated to be as high as 17–45% of the general population in Western countries ^2^, while among Asian populations it is reported to be between 15 and 20% ^3^. A progressive form of NAFLD called nonalcoholic steatohepatitis (NASH) can further progress to liver cirrhosis and hepatocellular carcinoma (HCC) ^4^. Recent studies have found that HCC may complicate non-cirrhotic NAFLD with or without fibrosis ^5^. In addition, NASH patients run an increased risk of cardiovascular mortality as a result of the metabolic risk factors that are common to both NAFLD and cardiovascular disease ^6^.

Major risk factors of NAFLD include a sedentary lifestyle and diet with poor nutrition ^2, 4, 7^. Other factors that influence the development of NAFLD include age, being a man between the ages of 40–65 years and Hispanic ethnicity ^1, 8– 11^. In addition, insulin resistance (IR), metabolic syndrome (MS) and type 2 diabetes mellitus (T2DM) are considered to increase the risk of NAFLD ^2^. NAFLD is closely associated with T2DM, and therefore T2DM is used as a determinant for the presence and severity of NAFLD ^12, 13^. Various studies have demonstrated that NAFLD is more prevalent in men, elderly populations, and post-menopausal women ^14– 17^.

Gender differences as a risk factor for NAFLD still need to be fully understood. There is controversy regarding gender and NAFLD; some studies claim that various gender-specific mechanisms, such as the effect of sex hormones and differences in lifestyles and physiology, have an influence on the prevalence of NAFLD ^15^. In addition, a number of studies report NAFLD as being more frequently detected in men than women ^8, 10, 14, 18, 19^. However, there are also some studies, both from Western and Asian populations, that suggest that the disease is generally more common in women ^9, 11, 20^.

Understanding the association between gender differences and NAFLD will allow us to target specific groups to improve health promotion and disease prevention activity, as well as provide proper treatment strategies in order to reduce the rates of morbidity and mortality associated with NAFLD and its associated pathologies. Therefore, this study investigated the gender differences in the prevalence of NAFLD from the general population of Northeast Thailand.

## Methods

This was a population-based cross-sectional study that retrieved the data from the baseline survey of an ongoing cohort research project called the Cholangiocarcinoma Screening and Care Program (CASCAP, www.cascap.in.th) ^21^. Data was retrieved between March 2013 and September 2015, and this project enrolled 65,571 Thai participants in Northeast Thailand who had at least one of the following risk factors: (1) were 40 years old or older; (2) had a previous infection with the liver fluke parasite; (3) had been treated with the chemotherapeutic drug, praziquantel; or (4) consumed raw or undercooked freshwater fish. In accordance with the CASCAP protocol, participants gave written informed consent and completed a baseline survey form. The standardized self-administered questionnaire included socio-demographic information including gender, behavioral factors, such as smoking status and alcohol consumption that can be classified into 2 categories as follows: 0=never, 1=yes, current or previous, and previous or current illnesses. After completion of the baseline survey, participants underwent hepatobiliary ultrasonography (US) performed by board-certified radiologists, who provided the participants with information on NAFLD. For the purpose of this study, subjects with alcohol consumption or those with incomplete information of US findings were excluded.

The primary outcome was the ultrasonographic diagnosis of NAFLD based on the presence of a diffuse increase of fine echoes in the liver parenchyma compared to the kidney or spleen parenchyma ^22^. This was performed after excluding other causes of liver disease, such as viral hepatitis B or C. Individuals were also excluded from the study if they had a history of current or past alcohol consumption ^15^. In addition, the protocol also classified the severity of NAFLD as follows: absent, mild, moderate or severe steatosis ^22, 23^. Finally, the participants were divided into those with and without NAFLD, according to the US results. Participants with absence of NAFLD were used as the comparison group for the study. The factor of interest in this study was gender.

Demographic characteristics and other information of the participants serving as covariates that could have an effect on the association between gender and NAFLD were accounted for in the statistical analysis. These included age, the presence of diabetes mellitus (DM) and other underlying diseases. Age was initially treated as a continuous variable based on the assumption of a linear relationship. For practical purposes, age was then categorized into six groups comprising: <45 years, 46–50 years, 51–55 years, 56–60 years, 61–65 years and more than 65 years. Other confounder factors included: the presence of DM and presence of other underlying diseases (ie HT, DLD, HD, CKD). These were dichotomous variables (0=never, 1=yes), and were also analyzed for the relationship with NAFLD.

### Data analysis

The characteristics of all enrolled participants were summarized by gender and the total number of study participants. All categorical variables were described by number and percentage of distributions. Continuous variables were expressed as means and standard deviation among male and female participants.

To answer the research questions, the overall prevalence of NAFLD, as well as NAFLD severity (mild, moderate, severe), and NAFLD combined with other abnormal US findings (periductal fibrosis (PDF), cirrhosis), was estimated separately for men and women. Univariate analysis using binomial regression was performed to explore the effect of gender and other clinical characteristics on NAFLD, ignoring the effect of other factors for better handling the covariates in a more sophisticated statistical modeling. In addition, we performed stratified analyses in pre-specific subgroups defined by age group and the presence of DM and other underlying diseases. The interaction of these stratified variables was investigated through a bivariate analysis performed by the Mentel-Haenszel extension of the chi-square test. Then, multivariable binomial regression was performed to quantify the effects of gender on NAFLD with the inclusion of age, DM, and other underlying diseases as covariates. These covariates were selected from variables based on the results of a bivariate analysis of each variable with the p-value < 0.25, and a literature review in which an association with NAFLD was shown. The effect of gender on NAFLD was then obtained as adjusted prevalence differences to demonstrate how certain risk factors impact the reduction of the overall prevalence of NAFLD and adjusted odds ratio (ORs) together with their 95% confidence intervals (CI). All analyses were done using Stata 13.1 (Stata Corp, College Station, TX, USA). The significance level was set at 0.05 and all statistical tests were two-sided.

### Ethical statement

CASCAP was approved by Khon Kaen University Ethics Committee (HE551404), and was conducted according to the International Conference of Harmonization, Good Clinical Practice guidelines and the Declaration of Helsinki. The authors of the present study submitted a Data Analysis Plan Proposal to Khon Kaen University Ethics Committee in Human Research to request the data (approval number, HE591067).

## Results

**Figure 1.**
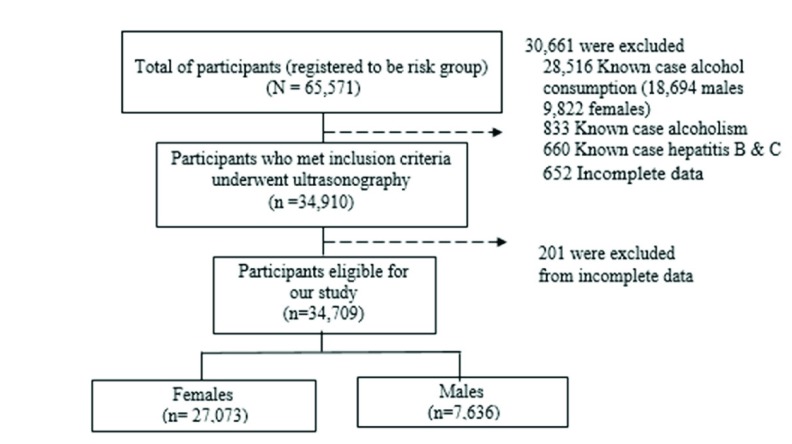
Flow of participants in the Cholangiocarcinoma Screening and Care Program.

A total of 65,571 participants living in northeastern Thailand agreed to participate in CASCAP during the study period as shown in Figure 1. We excluded 30,661 participants who were known to previously or currently consume alcohol, or to have viral hepatitis or alcoholism. Of the remaining participants, 201 were excluded because of incomplete data. Finally, a total of 34,709 participants were included for analysis.

Baseline characteristics of the study populations are shown in Table 1. Of 34,709 participants enrolled in this study, 27,073 were women (78.0%), while 7,636 were men (22.0%). Both were predominantly middle-aged with a mean age of 55.5±38.3 years old. There were similar characteristics between men and women, except that women were older (57.8 versus 54.3 years old, and had a lower proportion of being a current smoker or previously having smoked than men (1.0% vs. 18.7%).

**Table 1.** Baseline characteristics of the study population (n=34,709).

Characteristics	Female (n = 27,073)		Male (n=7,636)		Total (n = 34,709)	
	N	%	N	%	N	%
**Age at recruitment (years)**						
Less than 45	4,432	16.7	1,018	13.6	5,464	16.0
45 – 50	4,983	18.8	981	13.1	5,970	17.5
51 – 55	4,956	18.6	1,284	17.2	6,254	18.3
56 – 60	4,596	17.3	1,191	15.9	5,796	17.0
61 – 65	3,307	12.4	1,100	14.7	4,413	12.9
Greater than 65	4,306	16.2	1,914	25.5	6,226	18.3
Mean ±SD	57.8±42.3		54.3±37.1		55.5±38.3	
Median (Min: Max)	53.9 (40:98)		56.8 (40:99)		54.5 (40:99)	
**Education**						
No formal education	277	1.0	88	1.2	366	1.1
Primary school	21,122	78.2	5,968	78.3	27,137	78.1
Secondary school	1,753	6.5	504	6.5	2,261	6.5
Tertiary school	1,929	7.1	543	7.1	2,482	7.1
Collage	357	1.3	86	1.1	445	1.3
Under graduate	1,276	4.7	303	4.2	1,582	4.6
Post graduate	313	1.2	130	1.6	443	1.3
**Occupation**						
Unemployed	880	3.3	184	2.4	1,068	3.1
Farmer	21,868	80.9	6,314	82.9	28,236	81.3
Labor	1,194	4.4	354	4.7	1,552	4.5
Own business	810	3.0	227	3.0	1,040	3.0
Government/State enterprise	1,497	5.5	408	5.3	1,908	5.5
Others	789	2.9	131	1.7	920	2.6
**Smoking**						
No	26,551	99.0	6,133	81.3	32,752	95.1
Yes	269	1.0	1,415	18.7	1,684	4.9
**Underlying disease**						
No	21,817	80.6	6,231	81.1	27,839	81.0
Yes	5,256	19.4	1,405	18.9	6,870	19.0
**Diabetes mellitus**						
No	25,473	94.1	7,325	95.9	32,864	94.5
Yes	1,600	5.9	311	4.1	1,913	5.5
**Other underlying disease**						
No	25,028	92.5	7,160	93.7	32,251	92.7
Yes	2,045	7.5	476	6.3	2,526	7.3

Of 34,709 participants who underwent US, 7,584 had NAFLD; hence, the overall prevalence was 21.9% (95% CI: 21.4 to 22.3). The prevalence of NAFLD was 22.9% in women (95% CI: 22.5 to 23.5) and 18.3% in men (95% CI: 17.4 to 19.2) ( Table 2). Based on an absolute effect represented by the prevalence difference, the prevalence of NAFLD was significantly higher in women than men by 4.6% (95% CI: 3.6 to 5.6). Similarly, based on a relative effect represented by the OR, women were 1.3 times (1.3; 95% CI: 1.2 to 1.4) as likely to have NAFLD compared with men. After adjusting for effect of other covariates, the prevalence difference was 4.2% (95% CI: 3.2 to 5.2), but adjusted OR remained unchanged (1.3; 95% CI: 1.2 to 1.4).

**Table 2.** Prevalence difference and odds ratio demonstrating associations between gender and nonalcoholic fatty liver (n=34,709). DM, diabetes mellitus.

Factors	Female (n = 27,073)		Male (n = 7,636)		Prevalence difference	95% CI	p-value
	N	%	N	%			
Overall	6,186	22.9	1,398	18.3	4.6	3.6 to 5.6	<0.001
Crude	6,186	22.9	1,398	18.3	4.6	3.6 to 5.6	<0.001
Adjusted for age	6,186	NA ^*^	1,398	NA ^*^	4.5	3.6 to 5.5	<0.001
Adjusted for age and DM	6,186	36.4	1,398	33.4	4.2	3.2 to 5.2	<0.001
Adjusted for age DM and other underlying disease	6,186	34.2	1,398	23.6	4.2	3.2 to 5.2	<0.001
**Factors**	**N**	**%**	**N**	**%**	**Odds ratio**	**95% CI**	**p-value**
Crude	6,186	22.9	1,398	18.3	1.3	1.2 to 1.4	<0.001
Adjusted for age	6,186	NA ^*^	1,398	NA ^*^	1.3	1.2 to 1.4	<0.001
Adjusted for age and DM	6,186	36.4	1,398	33.4	1.3	1.2 to 1.4	<0.001
Adjusted for age DM and other underlying disease	6,186	34.2	1,398	23.6	1.3	1.2 to 1.4	<0.001

^*^ Both unadjusted and adjusted for age presence of DM and other underlying disease

The overall prevalence difference between gender was 4.6% (95%CI: 3.6 to 5.6), the prevalence difference in severity of ultrasonographic NAFLD with mild NAFLD was 3.8% (95%CI: 2.9 to 4.9) ( Table 3). The majority of participants with NAFLD were also found to have PDF, i.e. 1,143 participants had PDF out of the 7,584 participants with overall NAFLD. After combining NAFLD with the PDF, the increased prevalence in women remained, but the gender difference was smaller (1.1%; 95% CI: 0.6 to 1.3).

**Table 3.** Prevalence difference of nonalcoholic fatty liver between men and women according to steatotic grade and various combinations with other abnormalities in the ultrasound findings (n=34,709). US, ultrasound.

Steatosis grade	Total (n = 34,709)		Female (n = 27,073)		Male (n = 7,636)		Prevalence difference	95% CI	p-value
Overall	7,584	21.9	6,186	22.9	1,398	18.3	4.6	3.6 to 5.6	<0.001
Mild	5,843	16.8	4,773	17.6	1,054	13.8	3.8	2.9 to 4.9	<0.001
Moderate	1,657	4.8	1,336	4.9	319	4.2	0.7	0.6 to 0.8	<0.001
Severe	104	0.3	77	0.3	25	0.3	0.0	-0.5 to 1.0	0.549
**Combined with other** **abnormal US finding**	**Total** **(n = 34,709)**		**Female** **(n = 27,073)**		**Male** **(n = 7,636)**		**Prevalence** **difference**	**95% CI**	**p-value**
NAFLD with PDF	1,143	3.2	951	3.5	187	2.4	1.1	0.6 to 1.3	<0.001
NAFLD with PDF with cirrhosis	8	0.1	4	0.1	4	0.1	0.0	-1.3 to 1.3	1.000
NAFLD with cirrhosis	8	0.1	3	0.1	5	0.1	0.0	-1.4 to 1.4	1.000

**Figure 2.**
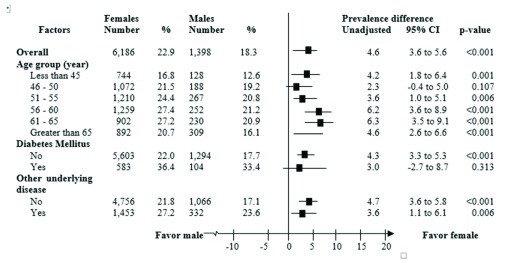
Prevalence differences of nonalcoholic fatty liver disease between women and men stratified by age group and underlying diseases.

Based on univariate analysis, prevalence differences stratified by age group demonstrated a noticeable pattern. That is, the difference in NAFLD prevalence tended to increase as the age increased. Moreover, while the overall prevalence difference was 4.6%, the stratified differences were 6.2% and 6.3% in the 56–60-year-old and 61–65-year-old age groups, respectively. The prevalence increased markedly in the age group up to 50 years, whereas both women and men at the age of 56–60 years have the highest prevalence of NAFLD (27.4% and 21.2%, respectively) ( Figure 2). Results also showed that the prevalence of NAFLD increased from 16.8% to 21.5% in women younger than 45 versus women aged 45–50 years, and then peaked at 27.4% in women aged 55–60 years. However, the prevalence differences of NAFLD decreased slightly in the ≥65-year-old age group.

**Figure 3.**
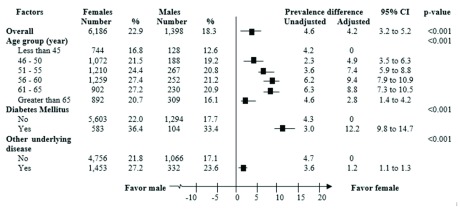
Adjusted prevalence differences of nonalcoholic fatty liver disease between women and men stratified by age group and underlying diseases. Each was adjusted for all others.

Results of multivariable analysis, the prevalence difference of NAFLD between men and women stratified by age group exhibited similar patterns, but with larger differences than were found in the univariate analysis. That is, while the overall adjusted prevalence difference was 4.2%, the stratified adjusted differences were 9.4% and 8.8% in the 56–60-year-old and 61–65-year-old age groups, respectively. The largest prevalence difference was observed among participants with DM or underlying diseases (12.2%; 95% CI: 9.8 to 14.7) ( Figure 3).

## Discussion

We investigated the inconsistencies in the literature regarding gender differences in NAFLD prevalence with a population-based study in Thailand. While many studies have indicated that NAFLD is more common in men than women ^10, 14, 18, 19^, others have suggested the opposite ^9, 11, 20^. Our study supported the findings of a higher NAFLD prevalence in women, with a 4.2% (95% CI: 3.2 to 5.2) prevalence, compared with men (18.3%; 95%CI: 17.4 to 19.2), even after adjusting for the effects of other covariates. In addition, we found that these differences increased with age, where the largest difference was found in the age group 56–60 years old (prevalence difference = 9.4%; 95% CI: 7.9 to 10.9). Moreover, the largest gender difference in NAFLD prevalence was also found among DM participants (12.2%; 95% CI: 9.8 to 14.7).

This study utilized data from CASCAP, in which most female participants were over 50 years old (64.5%), with a mean age of 57.8 ±42.3 years. The largest group (35.9%) of the over-50-year group being between 51–60 years old, and most were likely post-menopausal. Although the prevalence of NAFLD tended to be higher in women for every age group, the largest difference was found among the 56–60-year-old age group. This suggests that sex hormones might play a role in NAFLD ^16, 20^, for the younger age group. However, the current study revealed a smaller difference in NAFLD prevalence. This might be because NAFLD in men tends to occur earlier, especially in middle age (40–49 years) than in women (>50 years), which could lead to a male predominance in younger and middle-aged populations ^14^.

The gender differences on NAFLD prevalence became more pronounced as the age of the participants increased ^7, 24, 25^. The highest NAFLD rate was found in 56–60-year-olds. This suggested a correlation between NAFLD and the various major risk factors commonly found in older people, such as MS, obesity, DM, and dyslipidemia. Moreover, older patients are more likely to have advanced fibrosis, cirrhosis, and HCC when compared with middle-aged patients ^9^. Remarkably, the study found increased NAFLD prevalence in women in the transitional post-menopausal stage, especially among women aged 56–60 years. This statistic is consistent with a previous study that reported a 2 to 2.5 times higher prevalence in the 56–60-year-old age group compared with those aged less than 45 years ^26^. Our study also found that an increased prevalence of NAFLD in female participants persisted in the post-menopausal group, while the prevalence trend declined in those aged more than 65 years compared with premenopausal women.

### Age-gender interaction

The results of this study suggest that women are at higher risk of NAFLD than men. This has been attributed to natural changes in female physiology, such as IR, central obesity, adipose distribution and sex hormones ^20^. The gender differences in NAFLD observed in the study can be explained by the association of age and gender. Typically, younger-aged to middle-aged men tend to have a greater risk of acquiring NAFLD than women of the same age, as illustrated through an “inverted U-shaped curve”, in which the line begins to decline after the age of 50–60 years ^17^. Accordingly, premenopausal women have a relatively low prevalence of NAFLD; however, the prevalence increases after the age of 50 years, peaks at 60–69 years, and declines after age 70 years ^5, 16^. After the age of 50 years, the protective effects of higher estrogen levels in women during pre-menopause are markedly eliminated in the transitional post-menopausal period ^17, 26^. These associations between age and gender can be explained by natural changes in female physiology that increase the risk of IR, hyperlipidemia, and visceral fat accumulation, which are known as risk factors for the development of NAFLD ^27^. Estrogen is a powerful antioxidant that can inhibit hepatic stellate cell proliferation and fibrogenesis in experimental models ^16, 26^. These changes can reduce fatty acid oxidants, while increasing lipogenesis within the liver, which leads to a redistribution of subcutaneous fat and causes visceral fat accumulation ^16, 17, 27^. Therefore, changes in body fat distribution resulting from declining levels of estrogen, relatively higher androgen levels and greater distribution of hormone receptors can lead to increased risk of NAFLD in post-menopausal women ^16, 26, 28^. Subcutaneous and visceral compartmentalization of adipose tissue is influenced by age and gender ^20^. Visceral adipose tissue accumulates more rapidly with age and weight gain in men and post-menopausal women than in younger women ^20^. The NAFLD prevalence rate increases with age in all groups of younger to middle-aged men, and declines at the age of 50 to 60 years ^28^. However, NAFLD prevalence becomes comparable between men and women at the age of 60 years ^8, 14^. Our results confirm results showing that the interrelation between aging in premenopausal women and the development of NAFLD is strongly associated with changes in the level of estrogen-related sex hormones ^5^.

### Diabetes mellitus

Our data illustrate that T2DM patients had higher NAFLD prevalence compared with the general population ^12^. After adjusting for the effects of other covariates, it was found that NAFLD prevalence in those with T2DM was significantly higher (12.2%). Because a healthy population is included at the community level, DM is believed to be distributed randomly in men and women. Confounding effects of T2DM would be minimal. Previous studies reported an association between T2DM and NAFLD that was particularly pronounced in post-menopausal women >50 years old ^16^. This may be due to a decrease in estrogen in this group of women, which is a protective factor against DM ^29^. Moreover, IR in the muscle, liver, and adipose tissue is a characteristic feature of T2DM and NAFLD. It is characterized not only by higher insulin circulation levels, but also by higher hepatic gluconeogenesis, reduction of insulin clearance, and impaired glucose uptake by muscles, all of which lead to elevated plasma glucose concentrations ^30^. IR in adipose tissue can increase the release of free fatty acids and inflammatory cytokines ^31^. Transaminase levels increase in patients with NAFLD; however, this does not commonly occur in subjects who also have T2DM. Despite this, over the years, many patients with NAFLD have also been classified as having T2DM ^4^.

### Strengths and limitations

The study is a community-based study with a large sample size and a healthy population from the largest region of Thailand, the northeast region. In addition, the large size of the CASCAP database allows us to stratify the population by DM or non-DM, and to examine the interaction of different variables with adequate power. Second, the NAFLD diagnosis of all participants was performed by all board-certified radiologists. Finally, this study presented a strong link between gender and NAFLD presented with adjusted OR and absolute risk reduction (ARR). Using these statistical methods allowed us to properly measure the associations and determinants of certain health outcomes. It is important to note that, although the ARR varied according to event rates and the effects of other covariates, the adjusted OR remained unchanged. However, ARR is a valid index for healthcare providers because it demonstrates how certain risk factors impact the reduction of the overall prevalence of the disease.

Several limitations associated with the present study warrant mention. First, there were insufficient data to distinguish alcoholic fatty liver disease from NAFLD, so this differentiation was based on self-reported alcohol intake. Therefore, we excluded all participants with any history of alcohol intake, which affected the total number of male participants compared with that of females. However, when all participants were included back into the analysis, the prevalence of NAFLD in women remained higher than men in all age groups. Second, it should be considered that the database utilized in this study did not provide certain variables that may support a better determination of NAFLD progression; for example, anthropometric variables, such as BMI. Further studies are required to minimize these possibly distorted associations and allow generalization of these findings to other sampling populations.

## Conclusion

NAFLD is more likely to affect women than men, in particular among the population 56–60 years of age, which is the post-menopausal transitional period. This suggests that post-menopausal women should be concerned about metabolic disorders that are exacerbated by changing hormonal status. Monitoring and prevention by dietary control, behavioral changes, and exercise may play an important role in preventing diseases, including NAFLD. We strongly recommend and encourage Thai health professionals promotion of the development of NAFLD targeted screening and prevention programs focusing on post-menopausal women and DM risk groups.

## Data availability

The data referenced by this article are under copyright with the following copyright statement: Copyright: © 2017 Summart U et al.

Data associated with the article are available under the terms of the Creative Commons Zero "No rights reserved" data waiver (CC0 1.0 Public domain dedication). 

Researchers can request the CASCAP data by applying to the CASCAP Database Committee using a Data Analysis Plan Proposal. This can be found at http://www.cascap.in.th/damus/analysis_plan.php. More information can be requested from the corresponding author ( bandit@kku.ac.th) and information about research proposals can be found at https://cloud.cascap.in.th/article/research/index

## Supplementary material

Supplementary File 1: Do file to generate variables from the main CASCAP dataset used in the present study.

Supplementary File 2: Codes for variables from analysis.
