# Supplementary material for: Gender differences in the prevalence of nonalcoholic fatty liver disease in the Northeast of Thailand: A population-based cross-sectional study
Source: F1000Res. 2017 Oct 20;6:1630. Originally published 2017 Sep 4. [Version 2] doi: 10.12688/f1000research.12417.2 (PMC5645706; doi:10.12688/f1000research.12417.2)

Coding of all variables used in cca_01.csv file (Demographic information form: Enrollment

| id | Coding of the variable | Label | Values |
| --- | --- | --- | --- |
| 1 | f1v3 | Gender | 1=male 2=female |
| 2 | f1v4 | Education | 1=none 2=primary 3=secondary (M1-M3) 4= secondary (M4-M6)  5=Certificated 6= Bachelor 7=Higher than bachelor |
| 3 | f1v5 | Occupation | 1= Unemployed 2= Farmer 3= Labor  4= Own business 5= Government/State enterprise 6= Other |
| 4 | f1v10 | smoking | 0=no 1=yes or previous |
| 5 | f1v14a0 | Underlying disease | 0= no 1= yes |
| 6 | f1v14a3 | Diabetes mellitus | 0= no 1= yes |
| 7 | f1v14a4 | Other underlying disease | 0= no 1= yes |

Coding of all variables used in cca_02.csv file (Ultrasound Form)

| id | Coding of the variable | Label | Values |
| --- | --- | --- | --- |
| 1 | f2v2a1 | Parenchymal ECHO | 0= normal 1= abnormal |
| 2 | f2v2a1b1 | Fatty liver | 1a =Mild fatty liver, 1b=Moderate fatty liver, 1c = severe fatty liver |
| 3 | f2v2a1b2 | Peri ductal fibrosis (PDF) | 2a =PDF1, 2b = PDF2, 2c= PDF3 |
| 4 | f2v2a1b3 | Cirrhosis | 0= no 1= yes |

The primary outcome was US diagnosis of NAFLD based on the presence of a diffuse increase of fine echoes in the liver parenchyma compared with the kidney or spleen parenchyma.

1) In this study we generate this outcome NAFLD patients from f2v2a1b1 after excluding all of causes of fat accumulation in the liver such as infections with hepatitis viruses B or C and/or a history of current or past alcohol consumption.

2) NAFLD severity from f2v2a1b11a for mild NAFLD f2v2a1b11a for moderate NAFLD and f2v2a1b11c for severe NAFLD


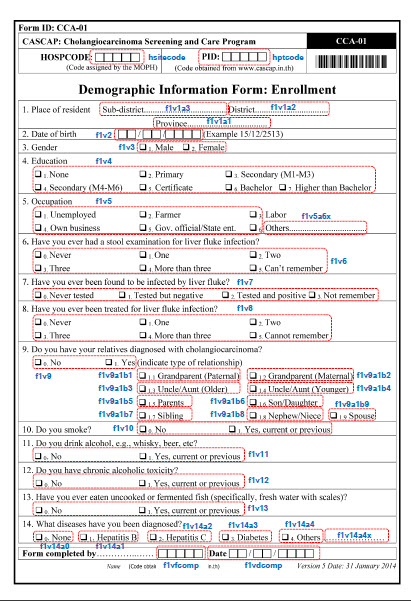


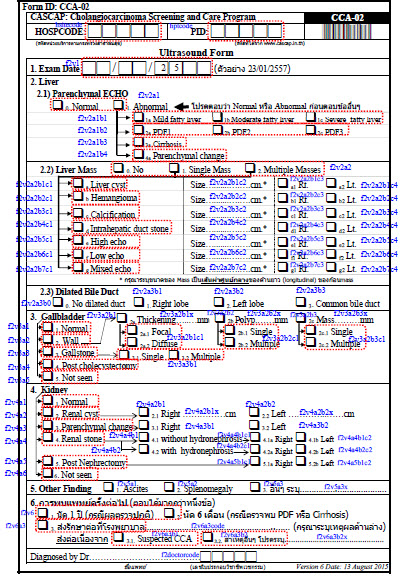

Supplement: Supplementary file 2 [file f1000research-6-14046-s0001.tgz › 9a6812c0-c4d9-47f0-bb51-6b63bf0e73bd.docx]
