# Supplementary material for: Gender differences in the prevalence of nonalcoholic fatty liver disease in the Northeast of Thailand: A population-based cross-sectional study
Source: F1000Res. 2017 Oct 20;6:1630. Originally published 2017 Sep 4. [Version 2] doi: 10.12688/f1000research.12417.2 (PMC5645706; doi:10.12688/f1000research.12417.2)
Supplement: Peer review history

# Peer review: Referee response for version 2

**DOI:** 10.5256/f1000research.14046.r27159

**Published:** 2017-11-27

**Authors:** Penprapa Siviroj, Mathuramat Seesen

**Affiliations:** 1 Faculty of Medicine, Chiang Mai University, Chiang Mai University, Thailand; 2 Faculty of Medicine, Chiang Mai University, Chiang Mai, Thailand

There is no doubt in the methodology and result of this research, but we are concerned about the conclusion which was pointed out that “We strongly recommend and encourage Thai health professionals promotion of the development of NAFLD targeted screening and prevention programs focusing on post-menopausal women and DM risk groups.” This research surveyed a very specific population which does not represent Thai population. There was no information supporting that the prevalence of NAFLD in people who have never had liver fluke parasite infection is similar to the one from this study group. We are serious about this point because it could lead to a wrong policy.

We have read this submission. We believe that we have an appropriate level of expertise to confirm that it is of an acceptable scientific standard, however we have significant reservations, as outlined above.

# Peer review: Referee response for version 2

**DOI:** 10.5256/f1000research.14046.r27160

**Published:** 2017-10-23

**Authors:** Nirun Intarut

**Affiliations:** 1 Clinical Epidemiology Unit, Faculty of Medicine, Mahasarakham University, Maha Sarakham, Thailand

I have no further comments.

I have read this submission. I believe that I have an appropriate level of expertise to confirm that it is of an acceptable scientific standard.

# Peer review: Referee response for version 2

**DOI:** 10.5256/f1000research.14046.r27158

**Published:** 2017-10-23

**Authors:** Matthew Kelly

**Affiliations:** 1 Department of Global Health, Research School of Population Health, Australian National University, Canberra, ACT, Australia

I have no further comments

I have read this submission. I believe that I have an appropriate level of expertise to confirm that it is of an acceptable scientific standard.

# Peer review: Referee response for version 1

**DOI:** 10.5256/f1000research.13446.r26831

**Published:** 2017-10-17

**Authors:** Nirun Intarut

**Affiliations:** 1 Clinical Epidemiology Unit, Faculty of Medicine, Mahasarakham University, Maha Sarakham, Thailand

This study aims to investigate the difference of NAFLD among gender. The author described the methodology results, and discussion clearly, but there have some point that need to clarify.

 The author mentioned in the Introduction part “Various studies have demonstrated that NAFLD is more prevalent in men, elderly populations, and post-menopausal women 14”. Please add more evidence in this sentence!

 Methods

- add more details of adjusted prevalence estimation, why the author use this methods for prevalence estimation. (according to the Figure 3)
- the author described this is a population-based cross-sectional study that was represented to (Northeast of Thailand population). Do they try to use weighted analysis?
- This wording might not be a reason for the analysis “ignoring  the effect of other factors for better handling the covariates in a more sophisticated statistical modeling”

 

 Results

- Table 1, No definition of “Underlying disease” and “Other underlying disease”, so please clarify these terms.
- Table 2, Please specify “age presence of DM” in the methods
  For odds ratio or adjusted prevalence estimation, no reason was mentioned “why do the author select only age, DM or other underlying to be in the model”
- Figure 2 and 3, please check “Other disease”, this is might be “Other underlying disease”.
- Figure 3, The graph presented sounds, but it might be unclear for interpretation. For example, after accounted for the possible confounder, the odds of DM was increased risk to NAFLD 4 times of crude OR.
  Moreover, for the other disease, the adjusted OR was lower than crude OR about 3 times. This is means that if people having some other diseases, they tended to having lower NAFLD. But we don’t know what a disease they have!

 

 Discussion

- Please add more details for others age group. Based on the Figures 2 and 3, the author mentioned only age 56-60. However, among 61-65 was greater risk to NAFLD as well.

I have read this submission. I believe that I have an appropriate level of expertise to confirm that it is of an acceptable scientific standard.

# Peer review: Referee response for version 1

**DOI:** 10.5256/f1000research.13446.r26390

**Published:** 2017-10-16

**Authors:** Matthew Kelly

**Affiliations:** 1 Department of Global Health, Research School of Population Health, Australian National University, Canberra, ACT, Australia

This paper was overall well presented and argued. The introduction is clear and makes a good rationale for the analyses in testing a contested area of the literature in gender differences in NAFLD. There are several points in the methods and discussion that could be made clearer however.

**Methods**

 Firstly the description of the study population is inadequate. Who the cohort are in terms of age and gender is clear. But we need to know how and why the cohort was recruited. It does not diminish the importance of the findings if cohort participants are not representative of the general population but it is still necessary to explain how they are different. Were they recruited because they were at particular risk of certain health conditions? Or just from the general population? How were they contacted? This will have implications in the discussion of your findings. 

 Also there are several references to those who consumed alcohol being excluded from the analysis. But there is no explanation of how this alcohol status was determined, and whether there was a threshold for alcohol consumption over the lifecourse. That is, are even occasional social drinkers excluded?

 The NAFLD itself was diagnosed using ultrasonography. But what about the other health conditions included in the analysis? Was diabetes also doctor diagnosed, or self-reported. And 'other underlying diseases are mentioned. What are they and how were they diagnosed?

 In summary we need to know what questions were included in the baseline questionnaire that led to these categorizations.

**Results**

 The results are well presented overall. There is one point that was a little unclear. In the 4th paragraph the authors mention interaction of the NAFLD with periductal fibrosis. No mention was made of this condition in the Methods. I am unclear what the importance of this interaction is or why it was mentioned. 

**Discussion**

 This section is also well set out and argued. The main point missing here is again who the cohort are, and whether the prevalence figures reported are applicable to the general population. I will state again here that not being nationally representative does not reduce the value of the findings. But it needs to be mentioned. 

 Lastly a minor point in the discussion. In the 'Age-gender interaction' section the authors state that '..NAFLD becomes comparable between men and women at the age of 60 years.' This does not appear to be true from the results. This point could maybe be explained more clearly.

I have read this submission. I believe that I have an appropriate level of expertise to confirm that it is of an acceptable scientific standard.

# Response

**Published:** 2017-10-16

**Authors:** Ueamporn Summart

**Affiliations:** Faculty of Public Health, Khon Kaen University, Khon Kaen, Thailand

Thanks for all of your help and suggestion for my paper. I promise to approve my paper as possible as I can and also remember all of your suggestions and comments to improve my new manuscripts.

 Best regards

 Ueamporn

# Peer review: Referee response for version 1

**DOI:** 10.5256/f1000research.13446.r25669

**Published:** 2017-10-04

**Authors:** Penprapa Siviroj, Mathuramat Seesen

**Affiliations:** 1 Faculty of Medicine, Chiang Mai University, Chiang Mai University, Thailand; 2 Faculty of Medicine, Chiang Mai University, Chiang Mai, Thailand

I would criticize and recommend the following:

**Introduction**

- The review literature about Nonalcoholic fatty liver disease is not up to date.
  Example: Younossi *et al*., 2016 ^1^

**Methods**

- The researcher should report the number of participants who were enrolled and excluded.
- The word “participants” in the second paragraph of page 3 is wrong.
- If there were comparison groups, the researcher should explain the purpose of the comparison group which was mentioned in the second paragraph of page 3.
- The researcher should explain what “other underlying disease” is.
- Figure 1 (Flow of participants) should be written with inclusion criteria before exclusion criteria. 

**Results and discussion**

- What is the purpose of reporting educational level and occupation in table 1? Were these factors used for adjusting the result?
- The age category should be similar in every part of the paper.
- In the discussion, the researcher categorized into groups ranging 10 years each (the prevalence increases after the age of 50 years, peaks at 60–69 years) but in the methods the age was categorized into groups ranging 5 years each.
- In the discussion, it was reported the prevalence of NAFLD declines after age 70, but in the results table reported the prevalence of age greater than 65.

**Conclusion**

- This research studied only prevalence of NAFLD which is only one of many kinds of metabolic disorder, so it is not appropriate to conclude that “post- menopausal women should be concerned about metabolic disorders”.
- This research surveyed a very specific population, so it is not appropriate to recommend NALFD screening and prevention programs focusing on post-menopausal women and DM risk groups because the prevalence of NAFLD in normal population might be different from this population.

We have read this submission. We believe that we have an appropriate level of expertise to state that we do not consider it to be of an acceptable scientific standard, for reasons outlined above.

## References

[1] YounossiZMKoenigABAbdelatifDFazelYHenryLWymerM: Global epidemiology of nonalcoholic fatty liver disease-Meta-analytic assessment of prevalence, incidence, and outcomes. *Hepatology*.2016;64(1) : 10.1002/hep.28431 73-84 10.1002/hep.28431 26707365
